# Supplementary material for: Bioactive Compound Diversity in a Wide Panel of Sweet Potato (Ipomoea batatas L.) Cultivars: A Resource for Nutritional Food Development
Source: Metabolites. 2024 Sep 26;14(10):523. doi: 10.3390/metabo14100523 (PMC11509433; doi:10.3390/metabo14100523)
Supplement: Supplementary file 1 [file metabolites-14-00523-s001.zip › FiguresS1andS2.pdf]

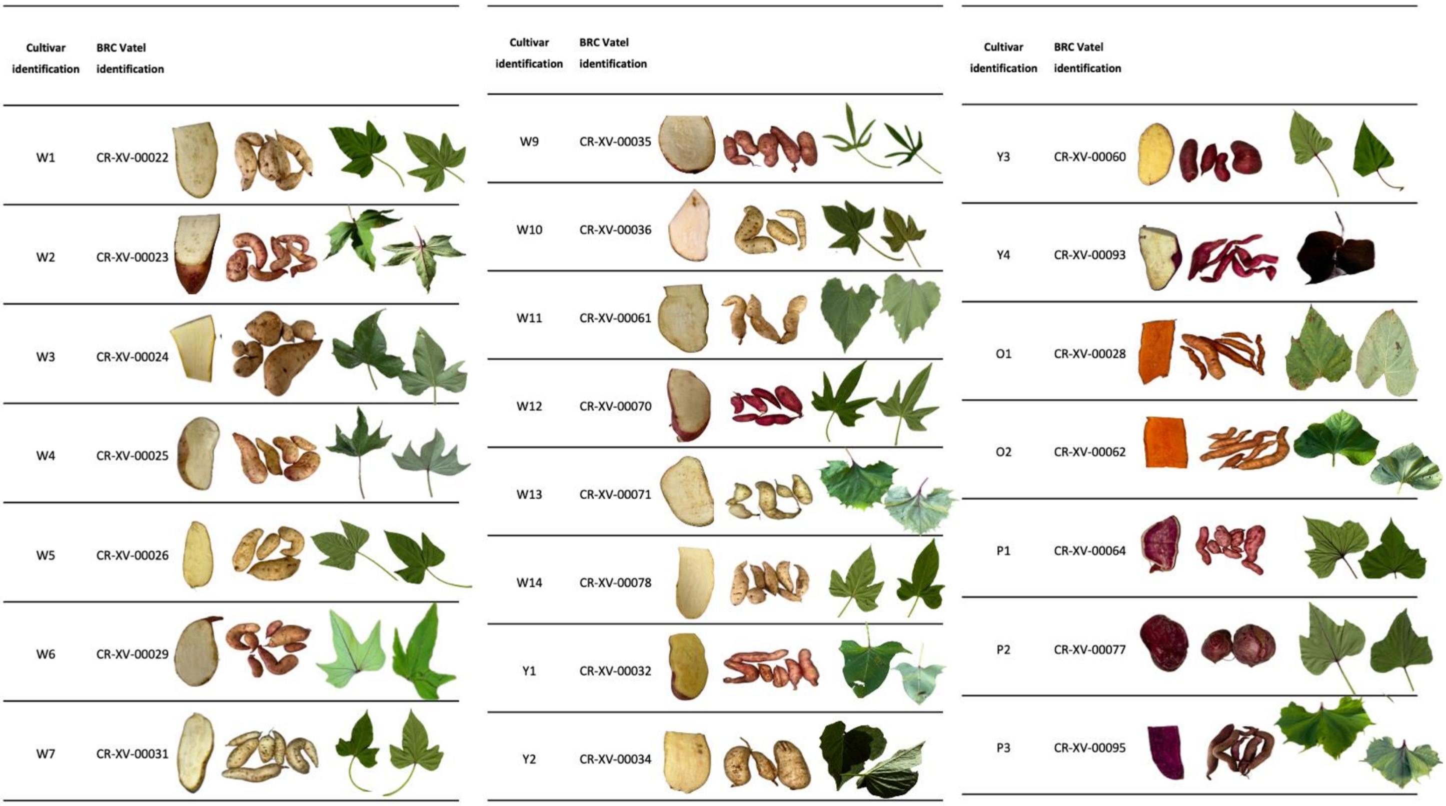

Figure S1. Phenotypic traits of roots and leaves for the 22 sweet potato cultivars from the Vatel BRC.

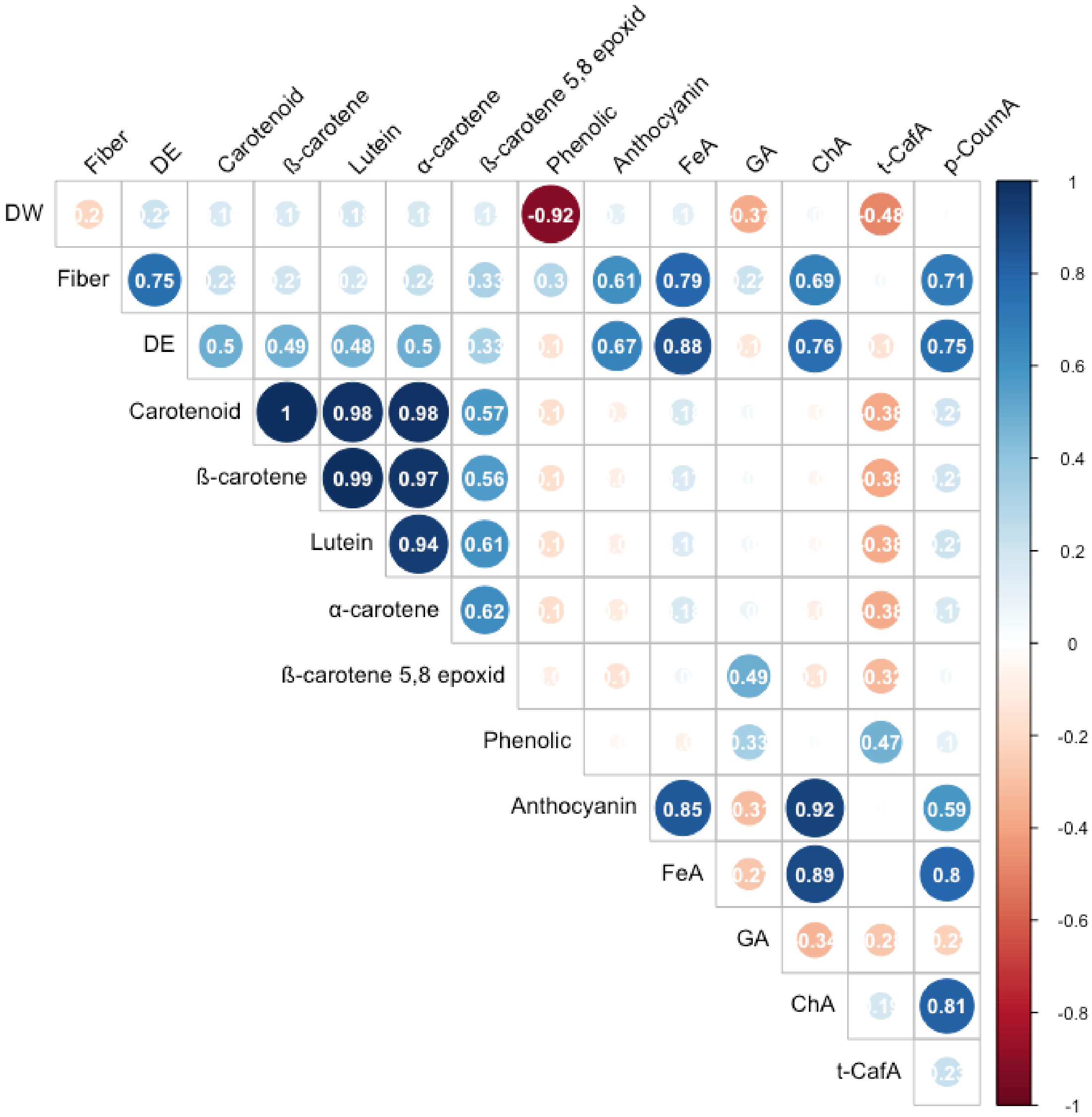

Figure S2. Correlation matrix with coefficients between contents of bioactive compounds for the 22 cultivars.
